# Reintroduction of Gluten Following Flour Transamidation in Adult Celiac Patients: A Randomized, Controlled Clinical Study

**DOI:** 10.1155/2012/329150

**Published:** 2012-07-31

**Authors:** Giuseppe Mazzarella, Virginia M. Salvati, Gaetano Iaquinto, Rosita Stefanile, Federica Capobianco, Diomira Luongo, Paolo Bergamo, Francesco Maurano, Nicola Giardullo, Basilio Malamisura, Mauro Rossi

**Affiliations:** ^1^Institute of Food Sciences, CNR, 83100 Avellino, Italy; ^2^Center for Coeliac Disease S. Maria dell'Olmo Hospital, 84013 Cava de' Tirreni Salerno, Italy; ^3^Gastroenterology Department, San G. Moscati Hospital, 83100 Avellino, Italy; ^4^IPALC Research & Development, 83040 Frigento, AV, Italy

## Abstract

A lifelong gluten-free diet (GFD) is mandatory for celiac disease (CD) but has poor compliance, justifying novel strategies. We found that wheat flour transamidation inhibited IFN-**γ** secretion by intestinal T cells from CD patients. Herein, the primary endpoint was to evaluate the ability of transamidated gluten to maintain GFD CD patients in clinical remission. Secondary endpoints were efficacy in prevention of the inflammatory response and safety at the kidney level, where reaction products are metabolized. In a randomized single blinded, controlled 90-day trial, 47 GFD CD patients received 3.7 g/day of gluten from nontransamidated (12) or transamidated (35) flour. On day 15, 75% and 37% of patients in the control and experimental groups, respectively, showed clinical relapse (*P* = 0.04) whereas intestinal permeability was mainly altered in the control group (50% versus 20%, *P* = 0.06). On day 90, 0 controls and 14 patients in the experimental group completed the challenge with no variation of antitransglutaminase IgA (*P* = 0.63), Marsh-Oberhuber grading (*P* = 0.08), or intestinal IFN-**γ** mRNA (*P* > 0.05). Creatinine clearance did not vary after 90 days of treatment (*P* = 0.46). In conclusion, transamidated gluten reduced the number of clinical relapses in challenged patients with no changes of baseline values for serological/mucosal CD markers and an unaltered kidney function.

## 1. Introduction

Celiac disease (CD) is caused by the ingestion of wheat gluten and related prolamins in genetically predisposed subjects [1], influencing 1% of population in developed countries [2, 3]. Currently, a lifelong gluten-free diet (GFD) is mandatory to alleviate the symptoms of CD and to normalize the antibodies in the intestinal mucosa [4]. Recovery is often observed after only a few weeks on a GFD [5]. CD is mainly characterized by the activation of intestinal gluten-specific CD4^+^ T cells [6]. In particular, gluten becomes a better antigen following its deamidation, which is catalyzed by tissue transglutaminase (tTG) [7]. Furthermore, proline residues protect against digestive proteolysis and direct tTG-mediated deamidation of glutamines [8]. A noteworthy finding was that gliadin can be cleaved by bacterial prolyl endopeptidases (PEPs) into short peptides that lose their activity [9]. Accordingly, oral PEP therapy has been proposed as a possible treatment [10, 11]. PEPs have also been evaluated as a technological tool for the preparation of detoxified gluten. Selected sourdough lactobacilli have specialized PEPs, and baked products from sourdough following 24 h of fermentation did not produce any alteration in intestinal permeability in 13 out of 17 patients [12]. A 60-day diet of baked goods made from hydrolyzed wheat flour, which was manufactured with sourdough lactobacilli and fungal proteases, was not toxic to patients with CD [13]. We tested a different enzymatic approach: the transamidation activity of food-grade microbial transglutaminase (mTG), a transamidase of the endo-*γ*-glutamine:*ε*-lysine transferase type [14]. We recently found that mTG exhibited the same site specificity as tTG but lacked the deamidase activity [15]. Most importantly, the transamidation of gliadin by treatment of wheat flour with mTG and lysine methyl ester (K-CH_3_) caused a dramatic downregulation of IFN-*γ* production *in vitro* in the intestinal T cells of CD patients [15].

In this first clinical study, we examined the safety of (K-CH_3_)-transamidated wheat flour in CD patients. The primary outcome measures included the appearance of clinical symptoms, by applying the gastrointestinal symptoms rating scale (GSRS) [16], and an altered intestinal permeability [17]. The second outcome was to evaluate the tolerance of treated flour by analyzing serum IgA antitissue transglutaminase (tTG) antibodies [18], changes in the Marsh degree of intestinal biopsies [19], and intestinal IFN-*γ* mRNA after 90 days [20]. In addition, by considering that the Q-K isopeptide, the final product of transamidation is largely metabolized in the kidney [21], we also determined creatinine clearance to monitor the integrity of renal function.

## 2. Materials and Methods

### 2.1. Patients and Study Design

**Figure 1:**
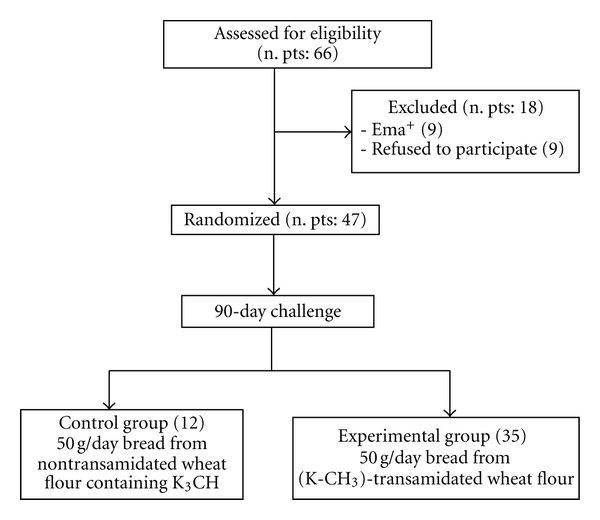
Flow chart of the study.

The study was a randomized, controlled clinical trial. 65 asymptomatic celiac patients on a GFD for almost two years were enrolled at two investigator sites: S. Maria Incoronata dell'Olmo Hospital, Cava de' Tirreni-SA, and the Gastroenterology Department of S. G. Moscati Hospital, Avellino. Nine of them declined participation after elucidation of the protocol study; another nine patients were excluded because of an antiendomysial antibodies (EMA)-positive test. Finally, 47 CD patients were enrolled for a 90-day trial. Their demographic characteristics, symptoms at diagnosis, and baseline laboratory data are reported in Table 1. Patients were randomized to receive 50 g/day of twice-baked bread slices produced from either nontransamidated wheat flour containing K-CH_3_ (control group, n.12) or (K-CH_3_)-transamidated wheat flour (experimental group, n.35). A simple randomization scheme was generated using a web site resource (http://www.randomization.com/). Patients were monitored for the appearance of clinical symptoms by using the gastrointestinal symptoms rating scale (GSRS) [16] and intestinal permeability [17] every 15 days throughout the study. On day 90, upper-gastrointestinal endoscopy and duodenal biopsies were performed to evaluate alterations.  Figure 1 is a flow chart of the study.

Patients with appearance of clinical symptoms related to gluten exposure or altered intestinal permeability were considered for withdrawn at any time. The following outcome measures were also considered for relapsed CD: changes in serum anti-tTG IgA antibodies [18], intestinal IFN-*γ* mRNA [20], and changes in the Marsh degree of examined intestinal biopsies [19].

### 2.2. Ethical Considerations

The study protocol was approved by the Ethical Committee of San G. Moscati Hospital (http://oss-sper-clin.agenziafarmaco.it/), OsSc registry n.06/09, trial n.234, 12/21/2007, and by the Ethical Committee ASL Salerno, OsSc registry n.318; trial n.118/AA.GG, 7/7/2009 in conformity to the provisions of the Declaration of Helsinki (as revised in Tokyo 2004). All participants gave their written informed consent.

### 2.3. Reagents

K-CH_3_ (99% purity) was purchased from Sisco Laboratories (Mumbai, India). Mono-dansylcadaverine (MDC) and ninhydrin were purchased from Sigma (St Louis, MO, USA). mTG (ACTIVA WM, 81–135 U/g) was provided by Ajinomoto Foods Europe, (Hamburg, Germany). All other reagents and solvents were available from Carlo Erba (Milan, Italy).

### 2.4. Biochemical Analyses of Flour

The transamidation activity on wheat flour was qualitatively estimated by using MDC. A concentration of 500 *μ*M MDC was added to a flour suspension in water. 8 U/g mTG was added, and the reaction was conducted for 2 h at room temperature. The protein fractions were then extracted with a modified Osborne procedure [22] and analyzed using 12% denaturing SDS-PAGE. Protein bands were visualized by UV and Coomassie R-250 blue staining. The enzyme reaction was quantitatively monitored using a modified ninhydrin assay [23]. The results were expressed as nmoles *α*-amino N/mg protein.

### 2.5. IFN-*γ* mRNA Analysis

RNA was extracted from the biopsies using the TRIzol reagent (Invitrogen, Milan, Italy). cDNA was prepared by reverse transcription. Real-time PCR was performed on the iCycler iQ (Bio-Rad Laboratories Inc, Hercules, CA, USA). The reaction conditions for 39 cycles were 95°C for 30 s, 56.4°C for 30 s, and 72°C for 40 s. Gene expression levels were calculated using the ΔΔCt method [24] and presented as fold changes after normalization to the L-32 housekeeping gene. The following primer sequences were used: L-32, forward 5′-CCTCAGCCCCTTGAAGC-3′; reverse 5′-GCCCTTGAATCTTCTACGAACC-3′; IFN-*γ*, forward 5′-TCAGCTCTGCATCGTTTTGG-3′, reverse 5′-GTTCCATTATCCGCTACATCTGAA-3′.

### 2.6. Enzyme Treatment

The large-scale quantitative transamidation of commercial wheat flour was conducted using food-grade mTG (8 U/g flour) and 20 mM K-CH_3_ for 2 h at 30°C, followed by centrifugation of the flour suspension. The recovered dough was used to manufacture the double-baked bread slices in gluten-free conditions; the control bread was similarly treated but without the addition of enzyme. A R5-sandwich ELISA analysis of the bread was performed by Imbiosis, SL (Madrid, Spain).

### 2.7. Intestinal Permeability Test

After an overnight fast, patients drank the test solution containing cellobiose (5 g), mannitol (2 g), and sucrose (40 g) dissolved in 100 mL of water (1500 mOsmol). All urine passed during the next five hours was collected into 25 *μ*M thiomersal. The mannitol and the cellobiose in the urine were measured using the method of Corcoran and Page [25] and Strobel et al. [17], respectively. Finally, the percent recovery ratio of cellobiose to mannitol (C/M) was calculated by assuming as upper limit of normality 0.037 [17].

### 2.8. Morphometric Analysis of Biopsy Specimens

Biopsies from the distal duodenum of patients were obtained during upper-GI endoscopy at time 0 and after 90 days of challenge. Two specimens were used for routine examination, whereas the others were stored in liquid nitrogen or embedded in the optimal cutting temperature (OCT) compound. Histology was performed according to a modified Oberhuber-Marsh classification [19]. Immunohistochemistry was performed on cryostat sections (5 *μ*m) fixed in acetone and stained according to the peroxidase-antiperoxidase (PAP) method. The sections were individually tested with monoclonal antibodies to CD3 (Dakopatts, Copenhagen, Denmark). The density of cells expressing CD3 in the intraepithelial compartment was determined by counting the number of stained cells as a percentage of 100 enterocytes.

### 2.9. Haematochemical Analyses and Creatinine Clearance

Blood samples were collected and analyzed for haemoglobin. The serum samples obtained at various times during the challenge were assayed for anti-tTG IgA antibodies using a commercial kit (Menarini Diagnostics srl, Firenze, Italy). Urine was collected for 24 h to determine the amount of creatinine that was removed from the blood per min (*C*_Cr_) according to standard protocols.

### 2.10. Statistical Analyses

CD-related gastrointestinal symptoms and intestinal permeability data were tabulated and compared via Fisher's exact test. The Mann Whitney test was used to compare patient ages, GFD periods, and anti-TG IgA titre. The Student's *t* test was used to analyze the ninhydrin reaction. Differences between the baseline and end results in the experimental group were determined by the Wilcoxon signed-rank test for the morphological, haematochemical, and serological analyses, by Chi-square test for the Marsh degree, and by the Kruskal-Wallis statistic and Dunn's Multiple Comparison Test for IFN-*γ* RNA. For all tests, the level *P* < 0.05 was selected to denote a significant difference.

## 3. Results

### 3.1. Transamidation of Flour Modified Both Gliadins and Glutenins without Altering Their Bread-Making Properties

**Figure 2:**
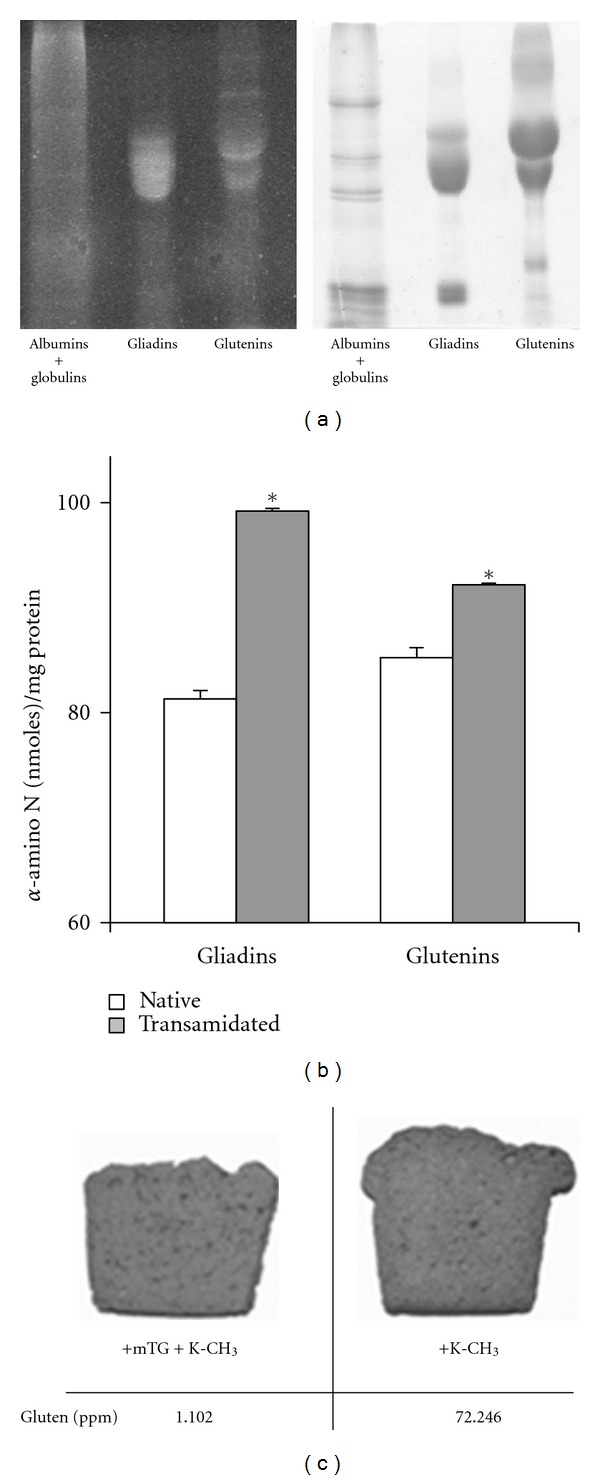
Activity of mTG in wheat flour. (a) SDS-PAGE of protein fractions following mTG-mediated transamidation of flour in the presence of MDC; the bands were visualized by UV (left) or Coomassie blue staining (right). (b) Quantification of lysine cross-linked to gliadins and glutenins following wheat flour transamidation with K-CH_3_; results are expressed as nmoles *α*-amino N/mg protein, and the statistical assessment was performed using the Student's *t* test; *: *P* < 0.05. (c) Baking features of dough following transamidation. Inset, gluten content in the sample breads as determined by R5-ELISA (Imbiosis, Madrid, Spain). These results are representative of five different experiments.

Wheat flour was incubated with mTG in the presence of MDC. As shown in Figure 2(a), the electrophoretic profiles indicated that both gliadins and glutenins, but not albumins and globulins, were substrates of mTG. The assessment of K-CH_3_ content found a significant increase in *α*-amino-N belonging to the cross-linked lysine in both gliadins and glutenins of the transamidated flour (Figure 2(b)). Then, we conducted a large-scale transamidation of the flour according to a previously established methodology [15]. The recovered experimental dough rose similarly to the control dough following the addition of yeast, and thus, it was possible to manufacture the bread used in the clinical trial (Figure 2(c)). Interestingly, the R5-ELISA indicated a drastic reduction in the levels of detectable protein, suggesting that following the binding of K-CH_3_, gliadin loses its cross-reactivity towards the R5 monoclonal antibody. The *in vitro* assessment of the immunostimulatory activity of gliadin isolated from transamidated flour confirmed the blockage of IFN-*γ* secretion in intestinal T-cell lines derived from CD patients (data not shown), as previously reported [15].

### 3.2. Assessment of Clinical Symptoms and Intestinal Permeability

47 CD patients on GFD were enrolled in the study. Demographic, clinical, and intestinal permeability and serological data are reported in Table 1; there were no differences between the two groups in the baseline values. Notably, a difference in the appearance of clinical symptoms was observed within two weeks of treatment (Table 2). The assessment of GSRS indicated that 9/12 (75%) and 13/35 (37%) of the patients in the control and experimental groups, respectively, had a clinical relapse (*P* = 0.04). It is noteworthy that 2/3 patients in controls had no symptoms and were asymptomatic at the time of diagnosis (Table 1). Baseline values of intestinal permeability were found to be normal in all patients, with the exception of one and two patients in the experimental and control groups, respectively (Table 1). After 15 days, 7/35 (20%) patients in the experimental and 6/12 (50%) in the control group showed an altered permeability (*P* = 0.06; Table 2).

In summary, 10/12 in controls and 14/35 patients in the experimental group were withdrawn from the study within 15 days for clinical relapse and/or altered permeability. In addition, two patients of the control and one of the experimental group dropped out for personal reasons.

Twenty patients in the experimental group continued the (K-CH_3_)-transamidated gluten challenge. Three more dropped out after 30 days, two for clinical relapse and altered C/M ratio, and one for personal reasons (Table 2). On day 90, 16 patients completed the study without developing clinical symptoms, but two more patients showed an altered C/M ratio at the end of the challenge (Table 2).

### 3.3. Analysis of Intestinal Mucosa after a 90-Day (K-CH_3_)-Transamidated Gluten Challenge

**Figure 3:**
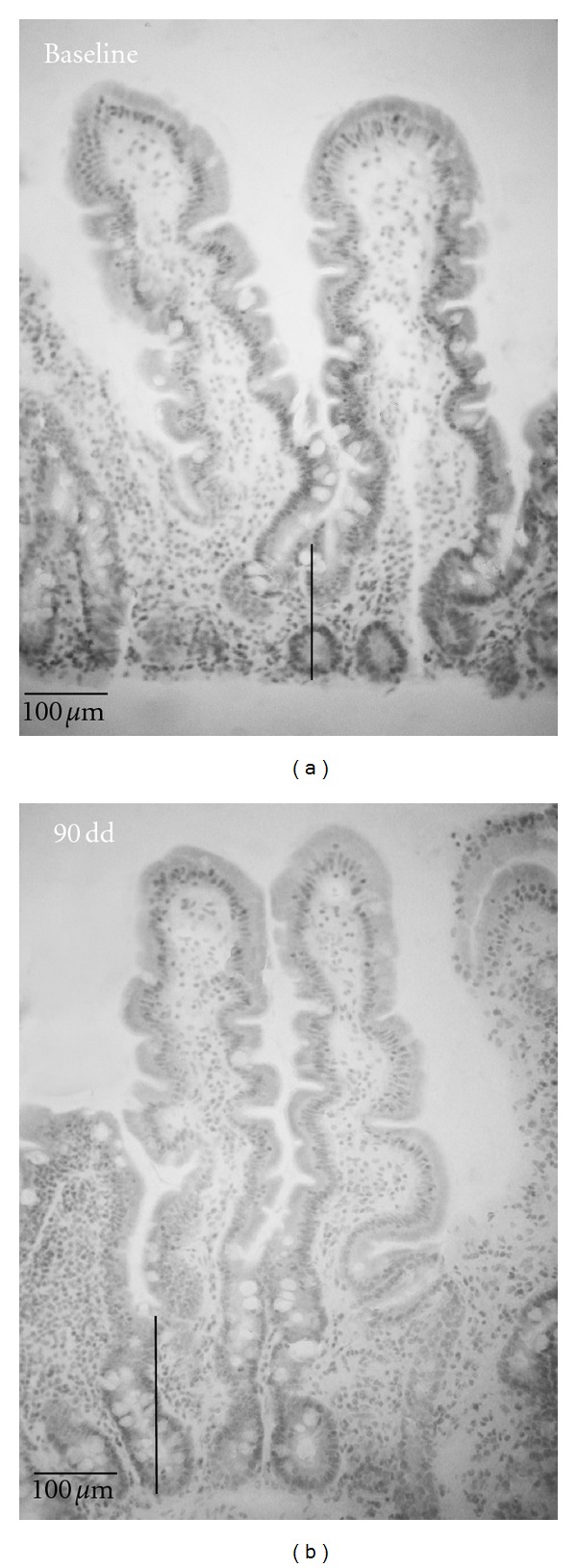
Histological appearance of intestinal mucosa from a CD subject at baseline (a) and following a 90-day (K-CH_3_)-transamidated gluten challenge (b). Lines indicate the crypt depths; original magnification: ×20.

**Figure 4:**
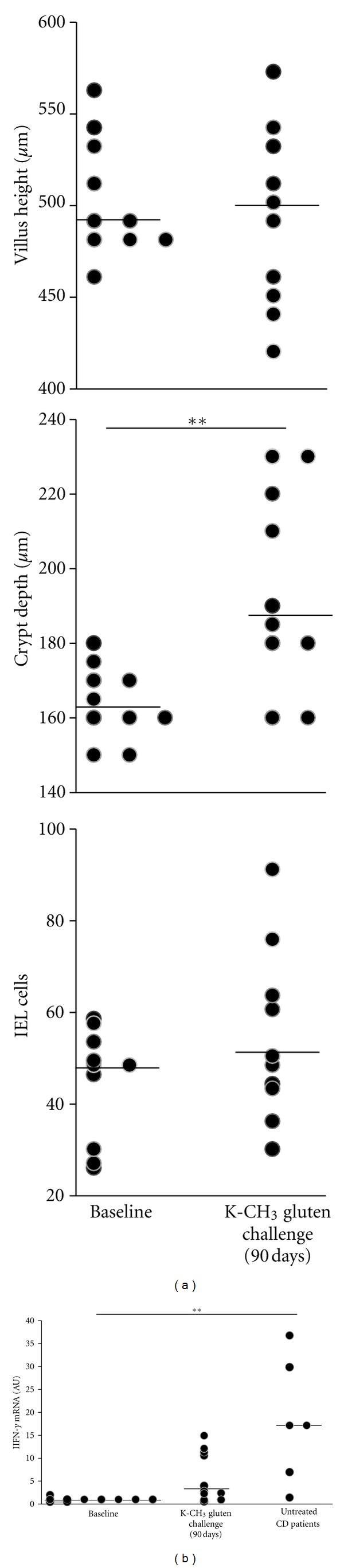
Assessment of intestinal mucosa following a 90-day (K-CH_3_)-transamidated gluten challenge. (a) Morphometric and immunohistochemical analyses of biopsy specimens from consenting patients (n.10) who completed the 90-day study without developing symptoms or altered permeability; the density of CD3^+^ cells in the intraepithelial compartment was determined by counting the number of stained cells as a percentage of 100 enterocytes; bars indicate medians, and the statistical evaluation of data was performed using the Wilcoxon signed-rank test. (b) IFN-*γ* mRNA levels in intestinal biopsies from patients in the experimental group (n.10) and from untreated CD patients (n.6) were evaluated by real-time PCR; the cytokine values were normalized to L-32 mRNA and are presented as fold change in gene expression (AU); bars indicate medians, and the Kruskal-Wallis statistic and Dunn's Multiple Comparison test were used to compare differences among groups (1) *: *P* < 0.05; **: *P* < 0.01.

Upper gastrointestinal endoscopy and biopsy specimens were obtained from 10/14 consenting patients who completed the 90-day study without developing symptoms or altered permeability. By evaluating the mucosal changes, we found that no subject developed villus subatrophy (Figure 3); moreover, the observed variation in the Marsh-Oberhuber grading was not found to be significant (Table 3). In particular, the statistical assessment did not indicate any difference in villus height with baseline values (*P* = 0.25) (Figure 4(a)). In contrast, the crypt depth values were found to be significantly different (Figures 3 and 4(a), *P* = 0.008). The analysis of IEL infiltration indicated no differences for CD3^+^ cells (*P* = 0.11). Next, the IFN-*γ* mRNA in the intestinal biopsies were evaluated. The results in Figure 4(b) showed a significant difference in the baseline values between the treated and untreated CD patients who are representing the positive control (*P* < 0.01). Interestingly, after 90 days of challenge with the transamidated gluten, the levels of IFN-*γ* transcripts were not significantly changed in the experimental group.

### 3.4. Laboratory Investigation following a 90-Day (K-CH_3_)-Transamidated Gluten Challenge

**Figure 5:**
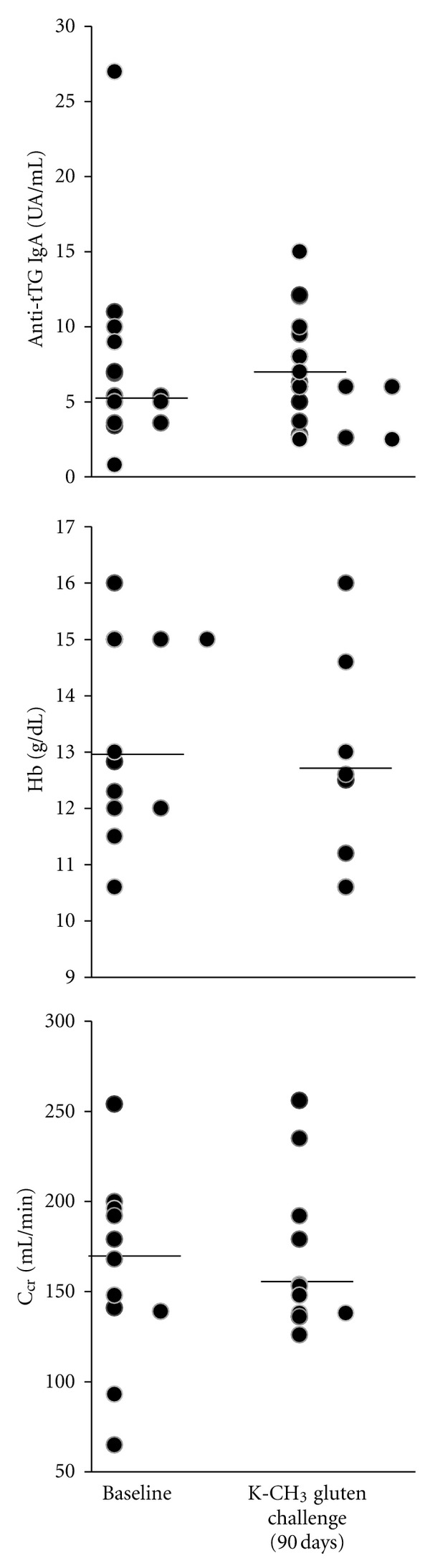
Laboratory investigation of patients in the experimental group. Anti-tTG IgA titre (UA/mL; n.17), haemoglobin content (g/dL; n.11) and creatinine clearance (mL/min; n.11) following a 90-day (K-CH_3_)-transamidated gluten challenge. Bars indicate medians. Statistical evaluation of the data was performed using theWilcoxon signed-rank test. The level *P* < 0.05 was selected to denote a significant difference.

Notably, the anti-tTG IgA end titre did not significantly increase in the patients undergoing a protracted transamidated gluten challenge (median, 5.7 versus 6.6, baseline versus post-challenge, *P* = 0.63, n.17; Figure 5). In addition, no substantial differences for haemoglobin were found (medians, 13.2 versus 13.0, baseline versus postchallenge, *P* = 0.64; n.11). As a measure of kidney function, creatinine clearance (*C*_Cr_) was evaluated and was found to be nonsignificantly changed (median, 168 versus 148, baseline versus postchallenge, *P* = 0.46; n.11). Similarly, no differences were reported for this parameter in the six normal subjects who underwent the same challenge with transamidated gluten (data not shown).

## 4. Discussion

In this trial, a protracted intake of gluten from wheat flour treated with mTG and K-CH_3_ was associated to a reduced number of relapses in challenged GFD CD patients.

The possibility of preventing immune activity against gluten has been underscored by the finding that the digestive resistance of gliadin may play a role in the pathogenesis of CD [26]. Gliadin can be cleaved by bacterial PEPs into short peptides that lose their immune activity [26, 27]. Accordingly, oral PEP therapy has been proposed as a possible treatment [10]. Recently, protein engineering has been exploited to improve PEP activity [28]. However, a very long fermentation is required to reduce the intolerance; therefore, it is still a challenge to prepare bread for CD patients [29]. 

We studied the transamidation activity of food-grade mTG, an alternative enzymatic approach to detoxify gluten [30]. Our previous experiments indicated that consequent to the transamidation with K-CH_3_, but not with lysine, the immune reactivity of gliadin was completely suppressed in intestinal T-cell lines isolated from CD patients [15].

It is widely accepted that both gliadins and glutenins are responsible for the toxicity in CD [31]. Therefore, it was an important finding that both protein types are substrates for mTG. Moreover, the dough from transamidated flour rose similarly to the control dough, which gave us the opportunity to produce normal slices of the double-baked bread used in the trial. Patients received 50 g/day of bread, corresponding to a 3.7 g daily gluten intake. This quantity is considered sufficient to reinduce the disease; in previous studies, 1–5 g gluten/day caused CD relapse on a clinical, laboratory and histological level both in children and in adults [32–34]. In a subsequent study, 50 mg gluten/day was considered the minimum dose required to produce measurable damage to the mucosa in CD patients [35]. Notably, the gluten content, detectable by R5-ELISA, drastically dropped following transamidation. We interpreted this data to mean that the enzymatic reaction masked the epitopes of the gliadins. In agreement with these observations, we detected increased levels of lysine moieties bound to transamidated gliadins and glutenins. Given the considerations described, a single-blinded, randomized, controlled trial was performed to verify the safety of the enzyme treatment in treated CD patients. We applied the GSRS, a disease-specific instrument, developed to evaluate common symptoms of gastrointestinal disorders [16]. We found that the dose was sufficient for producing a significant difference between the experimental and control groups in terms of clinical relapse after only 15 days. In fact, 37% and 75% of the experimental and control groups, respectively, exhibited clinical symptoms.

The intestinal permeability was investigated by the noninvasive C/M test [17] because, in CD, there is a decreased absorption of small molecules (mannitol) and a paradoxically increased absorption of large molecules (cellobiose). The test was valuable for monitoring dietary lapses in patients on a GFD [36]. Interestingly, although the C/M ratio was not significantly different between the experimental group and the control group after 15 days of treatment, the 90-day challenge showed that abnormal values essentially developed early, within 15 days, concomitantly with the timing of clinical relapse. In particular, only 2 patients in the experimental group dropped out later (Table 2), suggesting the existence of two distinct subsets of CD patients with different sensitivity to transamidated gluten. In line with these findings, we also registered unchanged haematological values after the 90-day challenge. On the other hand, these results indicated that our primary endpoint was only partially fulfilled.

The Q-K isopeptide, the final product of transamidation, is largely metabolized in the kidney, where *ε*-(*γ*-glutamyl)-lysine represents a substrate for *γ*-glutamylamine cyclotransferase (EC 2.3.2.4) [21]. The cleavage of the isopeptide bond by this enzyme results in the formation of free L-lysine and 5-oxoproline, which is metabolized to glutamic acid by 5-oxoprolinase. However, 5-oxoprolinase is an ATP-dependent enzyme, and ingestion of transamidated proteins would increase the consumption of ATP in the kidney, thus, potentially influencing renal function. Accordingly, we examined creatinine clearance in the experimental group and found that the end values were not different from the baseline values. Also, no differences were observed in the normal subjects undergoing the same challenge.

No variation was reported for the anti-tTG titres after 90 days, which are a good noninvasive indicator to assess the compliance in CD patients [18]. The morphometry and IEL counts of duodenal biopsies are considered hallmarks for a quantitative assessment of gluten-induced damage in CD [19]. Therefore, we compared the baseline and end values in 10 consenting patients who completed the 90-day challenge. The statistical evaluation of the morphological data highlighted a difference only in the crypt depth. Notably, the overall number of IELs, a very sensitive signal of mucosal damage, was unchanged. Accordingly, no significant changes in the Marsh-Oberhuber grade occurred in the experimental group. Taken together, the serological and morphological results confirmed that transamidated gluten was tolerated in this subset of CD patients. Finally, the IFN-*γ* mRNA levels in the intestinal biopsies at baseline and after the 90-day challenge did not show a significant difference, thus confirming our previous *in vitro* results [15].

## 5. Conclusions

The present study demonstrated that a protracted intake of gluten from wheat flour treated with microbial transglutaminase and lysine methyl ester was associated to a reduced number of relapses in challenged patients. Nevertheless, the enzyme reaction we described was not found sufficient in eradicating the gluten activity in all examined CD patients. Whether an upgrade of the transamidation reaction might be instrumental in blocking other immune components involved in the mucosal lesion is under investigation in our lab. Indeed, 19 out of the 94 glutamine residues in recombinant *α*-gliadin were identified as substrates for tTG [37]; this is a much higher number of substrates than that normally involved in generating DQ2/DQ8-restricted immunodominant epitopes. Therefore, novel studies should modify the reaction conditions to better address this issue.

## Figures and Tables

**Table 1:** Demographic data, clinical symptoms (GSRS) at diagnosis, and baseline laboratory investigations in 47 GFD CD patients.

	Group		*P* value^1^
	Control	Experimental
n. pts	12	35	
Age (years)^∗^	40 (23–50)	37 (18–53)	0.13
GFD (years)^∗^	3 (2–14)	8 (2–28)	0.07
Gender			
Female	9	24	
Male	3	11	1.00
GSRS			
Abdominal pain	10	20	0.16
Constipation syndrome	0	3	0.56
Diarrhea syndrome	4	18	0.33
Indigestion syndrome	1	1	0.44
Reflux syndrome	2	4	0.63
Total^2^	10	33	0.26
Nil	2	2	
C/M ratio > 0.037			
Baseline	2	1	0.15
Anti-tTG IgA titre (UA/mL)^∗^			
Baseline	6 (2.3–10.3)	5 (1.0–35.0)	0.35

^∗^ Median (range).

^1^GSRS, intestinal permeability data and gender distribution were compared via Fisher's exact test; the Mann Whitney test was used to analyze age, GFD and the anti-tTG IgA titre.

^2^Total number of patients manifesting at least one symptom.

**Table 2:** K-CH_3_-gluten challenge: clinical and intestinal permeability assessment.

Time of challenge (days)	15			30		60	90
	Group^∗^		*P* value^1^	Group		Group	Group
	ctr	exp		ctr	exp	exp	exp
n. pts	12	35		0	20	17	17
(A) C/M ratio > 0.037							
Baseline	2	1	0.15				
End	6	7	0.06	—	2	0	2
(B) GSRS							
Abdominal pain	7	13	0.31	—	2	0	0
Constipation syndrome	5	0	0.0005	—	0	0	1
Diarrhea syndrome	2	2	0.26	—	1	0	0
Indigestion syndrome	2	0	0.06	—	0	0	0
Reflux syndrome	0	0	1.00	—	0	0	0
Total^2^	9	13		—	0	0	0
Nil	3	22	0.04	—	18	17	16
Dropouts							
Withdrawn for (A) or (B)	10	14		—	2	0	—
Withdrawn for other reasons	2	1		—	1	0	

*ctr: control; exp: experimental.

^1^Fisher's exact test.

^2^Total number of patients manifesting at least one symptom.

**Table 3:** Biopsy analyses following a 90-day K-CH_3_-gluten challenge.

Marsh-Oberhauber	Baseline	90 days	*P* value^1^
Grade	n. pts	n. pts	
0	3	2	
1	7	4	
2	0	4	0.08

^1^Chi-square test.

## References

[1] Di Sabatino A, Corazza GR (2009). Coeliac disease. *The Lancet*.

[2] Rubio-Tapia A, Kyle RA, Kaplan EL (2009). Increased prevalence and mortality in undiagnosed celiac disease. *Gastroenterology*.

[3] Lohi S, Mustalahti K, Kaukinen K (2007). Increasing prevalence of coeliac disease over time. *Alimentary Pharmacology and Therapeutics*.

[4] Green PHR, Cellier C (2007). Medical progress: celiac disease. *The New England Journal of Medicine*.

[5] Murray JA, Watson T, Clearman B, Mitros F (2004). Effect of a gluten-free diet on gastrointestinal symptoms in celiac disease. *American Journal of Clinical Nutrition*.

[6] Sollid LM (2002). Coeliac disease: dissecting a complex inflammatory disorder. *Nature Reviews Immunology*.

[7] Molberg Ø, Mcadam SN, Körner R (1998). Tissue transglutaminase selectively modifies gliadin peptides that are recognized by gut-derived T cells in celiac disease. *Nature Medicine*.

[8] Hausch F, Shan L, Santiago NA, Gray GM, Khosla C (2002). Intestinal digestive resistance of immunodominant gliadin peptides. *American Journal of Physiology*.

[9] Shan L, Marti T, Sollid LM, Gray GM, Khosla C (2004). Comparative biochemical analysis of three bacterial prolyl endopeptidases: implications for coeliac sprue. *Biochemical Journal*.

[10] Cerf-Bensussan N, Matysiak-Budnilc T, Cellier C, Heyman M (2007). Oral proteases: a new approach to managing coeliac disease. *Gut*.

[11] Pyle GG, Paaso B, Anderson BE (2005). Effect of pretreatment of food gluten with prolyl endopeptidase on gluten-induced malabsorption in celiac sprue. *Clinical Gastroenterology and Hepatology*.

[12] Di Cagno R, De Angelis M, Auricchio S (2004). Sourdough bread made from wheat and nontoxic flours and started with selected lactobacilli is tolerated in celiac sprue patients. *Applied and Environmental Microbiology*.

[13] Greco L, Gobbetti M, Auricchio R (2011). Safety for patients with celiac disease of baked goods made of wheat flour hydrolyzed during food processing. *Clinical Gastroenterology and Hepatology*.

[14] Kanaji T, Ozaki H, Takao T (1993). Primary structure of microbial transglutaminase from Streptoverticillium sp. strain s-8112. *Journal of Biological Chemistry*.

[15] Gianfrani C, Siciliano RA, Facchiano AM (2007). Transamidation of wheat flour inhibits the response to gliadin of intestinal T cells in celiac disease. *Gastroenterology*.

[16] Revicki DA, Wood M, Wiklund I, Crawley J (1998). Reliability and validity of the gastrointestinal symptom rating scale in patients with gastroesophageal reflux disease. *Quality of Life Research*.

[17] Strobel S, Brydon WG, Ferguson A (1984). Cellobiose/mannitol sugar permeability test complements biopsy histopathology in clinical investigation of the jejunum. *Gut*.

[18] Sugai E, Nachman F, Váquez H (2010). Dynamics of celiac disease-specific serology after initiation of a gluten-free diet and use in the assessment of compliance with treatment. *Digestive and Liver Disease*.

[19] Oberhuber G, Granditsch G, Vogelsang H (1999). The histopathology of coeliac disease: time for a standardized report scheme for pathologists. *European Journal of Gastroenterology and Hepatology*.

[20] Nilsen EM, Jahnsen FL, Lundin KEA (1998). Gluten induces an intestinal cytokine response strongly dominated by interferon gamma in patients with celiac disease. *Gastroenterology*.

[21] Fink ML, Chung SI, Folk JE (1980). *γ*-Glutamylamine cyclotransferase: specificity toward *ε*-(L-*γ*-glutamyl)-L-lysine and related compounds. *Proceedings of the National Academy of Sciences of the United States of America*.

[22] Weegels PL, Hamer RJ, Schofield JD (1995). RP-HPLC and capillary electrophoresis of subunits from glutenin isolated by SDS and osborne fractionation. *Journal of Cereal Science*.

[23] Beckwith AC (1975). Direct estimation of lysine in cora meals by the ninhydrin color. *Journal of Agricultural and Food Chemistry*.

[24] Livak KJ, Schmittgen TD (2001). Analysis of relative gene expression data using real-time quantitative PCR and the 2^−ΔΔCT^ method. *Methods*.

[25] Corcoran AC, Page IH (1947). A method for the determination of mannitol in plasma and urine. *The Journal of Biological Chemistry*.

[26] ArentzHansen H, Mcadam SN, Molberg O (2002). Celiac lesion T cells recognize epitopes that cluster in regions of gliadins rich in proline residues. *Gastroenterology*.

[27] Marti T, Molberg Ø, Li Q, Gray GM, Khosla C, Sollid LM (2005). Prolyl endopeptidase-mediated destruction of T cell epitopes in whole gluten: chemical and immunological characterization. *Journal of Pharmacology and Experimental Therapeutics*.

[28] Ehren J, Govindarajan S, Morón B, Minshull J, Khosla C (2008). Protein engineering of improved prolyl endopeptidases for celiac sprue therapy. *Protein Engineering, Design and Selection*.

[29] Cabrera-Chávez F, Calderón de la Barca AM (2010). Trends in wheat technology and modification of gluten proteins for dietary treatment of coeliac disease patients. *Journal of Cereal Science*.

[30] Yokoyama K, Nio N, Kikuchi Y (2004). Properties and applications of microbial transglutaminase. *Applied Microbiology and Biotechnology*.

[31] Molberg Ø, Solheim Flaete NS, Jensen T (2003). Intestinal T-cell responses to high-molecular-weight glutenins in celiac disease. *Gastroenterology*.

[32] Laurin P, Wolving M, Fälth-Magnusson K (2002). Even small amounts of gluten cause relapse in children with celiac disease. *Journal of Pediatric Gastroenterology and Nutrition*.

[33] Jansson UHG, Gudjónsdóttir AH, Ryd W, Kristiansson B (2001). Two different doses of gluten show a dose-dependent response of enteropathy but not of serological markers during gluten challenge in children with coeliac disease. *Acta Paediatrica*.

[34] Montgomery AMP, Goka AKJ, Kumar PJ, Farthing MJG, Clark ML (1988). Low gluten diet in the treatment of adult coeliac disease: effect on jejunal morphology and serum anti-gluten antibodies. *Gut*.

[35] Catassi C, Fabiani E, Iacono G (2007). A prospective, double-blind, placebo-controlled trial to establish a safe gluten threshold for patients with celiac disease. *American Journal of Clinical Nutrition*.

[36] Hamilton I, Cobden I, Rothwell J, Axon ATR (1982). Intestinal permeability in coeliac disease: the response to gluten withdrawal and single-dose gluten challenge. *Gut*.

[37] Mazzeo MF, De Giulio B, Senger S, Rossi M, Malorni A, Siciliano RA (2003). Identification of transglutaminase-mediated deamidation sites in a recombinant *α*-gliadin by advanced mass-spectrometric methodologies. *Protein Science*.

